# Association of miR-34a with metastatic progression through the FoxO3a–SIRT1 regulatory axis in breast cancer

**DOI:** 10.55730/1300-0144.6166

**Published:** 2025-11-27

**Authors:** Banu ŞAHİN, İlker Batuhan BURAL, Hüseyin ÖZCAN, Sendegül YILDIRIM, Gamze TANRIÖVER, Şükran Burçak YOLDAŞ

**Affiliations:** 1 Faculty of Medicine, Akdeniz University, Antalya, Turkiye; 2 Department of Histology and Embryology, Faculty of Medicine, Ufuk University, Ankara, Turkiye; 3 Department of Histology and Embryology, Faculty of Medicine, Akdeniz University, Antalya, Turkiye; 4 Department of Medical Biology and Genetics, Faculty of Medicine, Akdeniz University, Antalya, Turkiye

**Keywords:** Breast cancer, metastasis, SIRT1, FoxO3a, miR-34a

## Abstract

**Background/aim:**

Breast cancer represents the foremost cause of cancer-related mortality among women worldwide and is characterized by a markedly high metastatic potential, contributing substantially to its clinical severity and poor prognosis. This study aimed to evaluate the interrelationship among silent information regulator (SIRT1), Forkhead box O3a (FoxO3a), and microRNA-34a (miR-34a), the latter of which promotes apoptosis by suppressing the proliferation, migration, and invasion of breast cancer cells. Specifically, we investigated the tumor microenvironment associated with metastatic progression of malignant primary tumors, the influence of nonmetastatic (benign) tumors, and the potential roles of these proteins in both primary tumor development and metastasis.

**Materials and methods:**

In this study, metastatic 4TLM and nonmetastatic 67NR breast cancer cell lines were used. These cell lines were orthotopically injected into the mammary fat pads of 8–10-week-old female Bagg Albino laboratory-bred strain c (BALB/c) mice. Mice were sacrificed 28 days postinjection, and primary tumors, lungs, and liver tissues were collected for analysis. Expression levels of SIRT1, FoxO3a, and miR-34a were evaluated using immunohistochemistry, Western blotting, and RT-PCR.

**Results:**

Expression levels of SIRT1 and FoxO3a were significantly higher in metastatic 4TLM tumors compared to the nonmetastatic 67NR group. Conversely, miR-34a expression was markedly higher in nonmetastatic tumors, whereas its level was reduced in metastatic tissues (p < 0.05). In metastatic tissues, SIRT1 expression remained elevated, whereas FoxO3a and miR-34a levels were significantly reduced (p < 0.05).

**Conclusion:**

SIRT1 may act as either a tumor suppressor or a tumor promoter, depending on cellular context, signaling pathways, and its specific molecular targets across cancer types. The elevated expression of SIRT1 in 67NR primary tumors suggests a tumor-suppressive role in nonmetastatic settings. However, the increased SIRT1 expression observed in metastatic regions indicates its potential role as a tumor promoter and modulator of the tumor microenvironment, possibly through FoxO3a and miR-34a signaling, thereby enhancing cellular proliferation and invasion.

## 1. Introduction

Cancer is a multifactorial and heterogeneous disease characterized by uncontrolled cellular proliferation, representing a major global health burden.^1^ Among various cancer types, breast cancer remains one of the most prevalent malignancies in women and ranks as the leading cause of cancer-related mortality worldwide.^2^ In Türkiye, breast cancer accounts for approximately 23.9% of all cancer diagnoses.^3^ Given its high incidence, elucidating the molecular mechanisms underlying breast cancer pathogenesis and identifying reliable biomarkers for early detection are critical for developing effective therapeutic strategies. One of the major challenges in breast cancer treatment is metastasis—the spread of malignant cells to distant organs through the blood or lymphatic systems—which markedly worsens prognosis and complicates clinical management [1]. The metastatic spread of breast cancer cells plays a pivotal role in disease progression and treatment outcomes. The metastatic cascade encompasses a series of complex molecular interactions and genetic alterations that profoundly affect disease progression and therapeutic response [2]. Accordingly, the molecular relationship between metastasis and tumor progression remains a central focus in cancer biology and therapeutic research.

The Forkhead box O (FOXO) family consists of transcription factors characterized by a conserved winged-helix DNA-binding domain—known as the FOX domain—that regulate genes involved in oxidative stress response, DNA repair, and cell survival [3]. Activation of FOXO factors has been shown to extend lifespan in model organisms such as *C. elegans* and *Drosophila* [4].

Conversely, sirtuins (SIRTs) constitute a highly conserved family of NAD^+^-dependent enzymes involved in cellular stress responses and lifespan regulation across various species [5]. Originating from the yeast gene silent information regulator 2 (*Sir2*), SIRTs share a conserved catalytic core and exhibit multiple enzymatic activities, including deacetylation, mono-ADP-ribosylation, and deacylation. Among them, SIRT1 has attracted significant interest due to its roles in aging, cellular metabolism, and tumorigenesis. Notably, SIRT1 modulates the transcriptional activity of FoxO3a and other FOXO proteins, thereby promoting cancer cell survival and invasiveness under stress conditions [6].

MicroRNAs (miRNAs) are small, noncoding RNA molecules, typically 22–25 nucleotides long, that regulate gene expression posttranscriptionally and play crucial roles in cancer development, progression, and therapeutic resistance. Among them, miR-34a is one of the most extensively studied tumor-suppressive miRNAs. Frequently downregulated in breast cancer and other malignancies, miR-34a suppresses cancer cell proliferation, migration, and invasion, while promoting apoptosis and increasing sensitivity to chemotherapy and radiotherapy. [7–10]. Mechanistically, miR-34a targets several oncogenes and signaling pathways, including *Notch1*, *SIRT1*, *BCL2*, and *CDK6*, thereby exerting broad antitumor effects [1,11]. Clinical studies have shown that reduced miR-34a expression in breast tumors correlates with higher histological grade, larger tumor size, lymph node metastasis, and poorer prognosis. In triple-negative breast cancer (TNBC), miR-34a regulates the FOXM1/eEF2K pathway and modulates drug resistance through *ABCB1* and *ABCG2*, underscoring its potential as both a diagnostic biomarker and therapeutic target [1,12].

The regulatory axis involving miR-34a, SIRT1, and FoxO3a plays a pivotal role in breast cancer metastasis. In our previous study, we demonstrated that SIRT1 enhances cancer cell survival and invasiveness by regulating FoxO3a-dependent target genes [3].

In TNBC tissues, SIRT1 is predominantly localized in the nucleus, whereas FoxO3a expression is enriched in the cytoplasm. Moreover, SIRT1 mRNA is highly expressed in metastatic tissues, and miRNAs—particularly miR-34a—suppress SIRT1 expression by directly targeting its mRNA. Specifically, miR-34a upregulates FoxO3a expression, which in turn induces apoptosis in breast cancer cells. Notably, miR-34a is transcriptionally regulated by the tumor suppressor p53. Mutations in p53, which are frequently observed in cancers, lead to miR-34a downregulation and a consequent reduction in FoxO3a expression. Importantly, miR-34a has been shown to inhibit SIRT1 activity, thereby inducing apoptosis and reducing cancer cell viability [12]. Despite these insights, research on the intersection of SIRT1, FoxO3a, and miR-34a remains limited.

In this study, we aimed to elucidate the role of miR-34a in regulating the FoxO3a signaling pathway during breast cancer metastasis. Using the murine model, we orthotopically injected metastatic 4T1 breast cancer cells into the mammary fat pads of female BALB/c mice. After 28 days, we assessed miR-34a expression and its influence on the FoxO3a signaling pathway in stage IV breast tumors using reverse transcription polymerase chain reaction (RT-PCR). The findings from this study advance our understanding of the molecular dynamics underlying miR-34a-mediated regulation in metastatic breast cancer and may contribute to the development of novel gene-targeted therapeutic strategies.

## 2. Materials and methods

### 2.1. Animal grouping, sample labeling, and cDNA preparation

This study utilized archived complementary DNA (cDNA) samples derived from a previous in vivo experiment involving 16 female BALB/c mice obtained from the Kobay Animal Laboratory (Ankara, Türkiye). Of these, eight mice were orthotopically implanted with metastatic tumor tissues derived from the 4TLM cell line, whereas the remaining eight received nonmetastatic tissues originating from the 67NR cell line. For the present analysis, stored cDNA samples previously extracted from primary tumor tissues were retrieved from the institutional biobank, where they had been preserved under optimal storage conditions [6]. Although the use of previously stored cDNA samples enabled a focused investigation of the core regulatory axis, we acknowledge that it may introduce variability in sample integrity. This limitation was mitigated by rigorously evaluating both the integrity and concentration of the cDNA samples prior to qRT-PCR. The expression level of the target molecule, miR-34a, was subsequently quantified using the RT-PCR. All experimental protocols were approved by the Local Ethics Committee for Animal Research of Akdeniz University (protocol no: 2016.09.02).

### 2.2. Experimental methodology and sample preparation

Prior to gene expression analysis, the integrity and concentration of the cDNA samples were assessed to ensure suitability for qRT-PCR. Amplification reactions were carried out using a commercial kit (FastStart Essential DNA Green Master; Qiagen GmbH, Hilden, Germany) in accordance with the manufacturer’s protocol. Specific primers targeting the genes of interest were selected based on previously published studies, and their sequences are presented in Table.

### 2.3. miR-34a expression analysis using RT-PCR

Quantification of miR-34a expression was carried out using the comparative cycle threshold (ΔΔCt) method.

Fluorescence data were obtained using SYBR Green I dye (Thermo Fisher Scientific, Waltham, MA, USA), which binds specifically to amplified DNA fragments, and cycle threshold (Ct) values were averaged across three technical replicates to ensure analytical reliability. Expression levels were normalized to an internal reference gene (18S rRNA), which served as the endogenous control. Primer–dimer formation and amplicon specificity were verified through melt-curve analysis. Following normalization, fold changes in gene expression were calculated using the 2^−ΔΔCt^ method, as described by Livak and Schmittgen (2001, *Methods 25: 402–408*), to determine relative differences in mRNA expression among experimental groups. The stability of the 18S rRNA endogenous control used for normalization was validated by consistent Ct values (SD < 0.5) across all experimental groups.

### 2.4. Data analysis and interpretation

At the conclusion of the study, miR-34a expression levels were compared between primary tumor tissues derived from orthotopic injections of metastatic 4TLM and nonmetastatic 67NR cell lines into the mammary fat pads of BALB/c mice. These findings were further interpreted in relation to previously reported FoxO3a gene expression profiles obtained from the same set of tumor tissues.

Statistical analyses were performed using SPSS Statistics software (IBM Corp., Armonk, NY, USA).

Continuous variables were expressed as mean ± standard deviation (SD) or median (interquartile range [IQR]), depending on the distribution pattern. Categorical variables were presented as frequencies and percentages. Associations between categorical variables were evaluated using Pearson’s chi-square test or Fisher’s exact test, as appropriate.

## 3. Results and discussion

The expression levels of FoxO3a, SIRT1, and miR-34a were assessed across different tumor tissue types to investigate their differential regulation under metastatic and nonmetastatic conditions.

**Figure 1:**
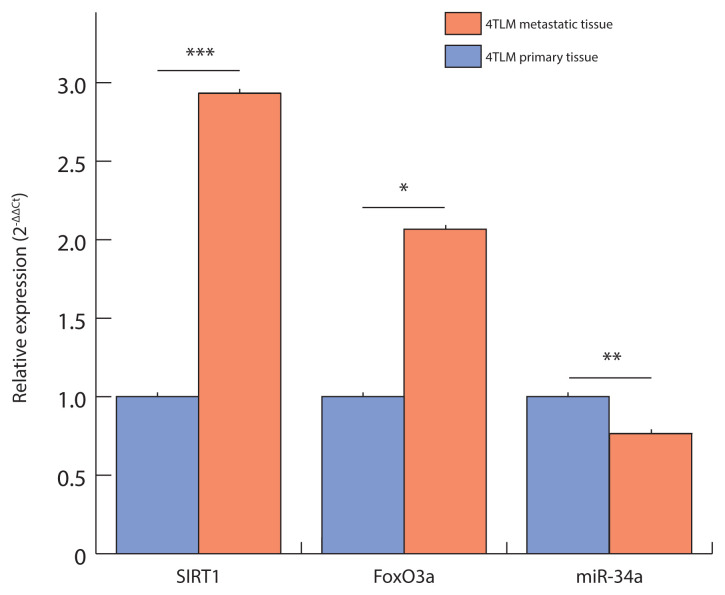
Grouped bar chart illustrating the relative gene expression (2^−ΔΔCt^) of FoxO3a, SIRT1, and miR-34a in 4TLM-derived primary versus metastatic tumor groups. Expression levels are presented as mean fold-change values relative to the primary tumor group (blue bars = 4TLM primary), using 18S rRNA as the reference gene for normalization (ΔCt) and 4TLM primary as the calibrator (ΔΔCt). Error bars represent the standard deviation (±SD) of eight biological replicates per group. Significant differences between primary and metastatic groups for each gene are indicated by asterisks (independent t-tests: ns = not significant; ^*^ p < 0.05; ^**^ p < 0.01; ^***^ p < 0.001).

As illustrated in Figure 1, FoxO3a and SIRT1 expression levels were significantly elevated in 4TLM-derived metastatic tumors compared to their corresponding primary tumors, whereas miR-34a levels were markedly reduced. Specifically, FoxO3a was upregulated approximately 2.1-fold (p = 0.02), SIRT1 2.9-fold (p = 0.0005), and miR-34a was downregulated to 0.7-fold (≈30% decrease; p = 0.007) in metastatic tissues. These statistically significant differences suggest that metastasis in the 4TLM model is associated with upregulation of FoxO3a and SIRT1 and downregulation of miR-34a.

**Figure 2:**
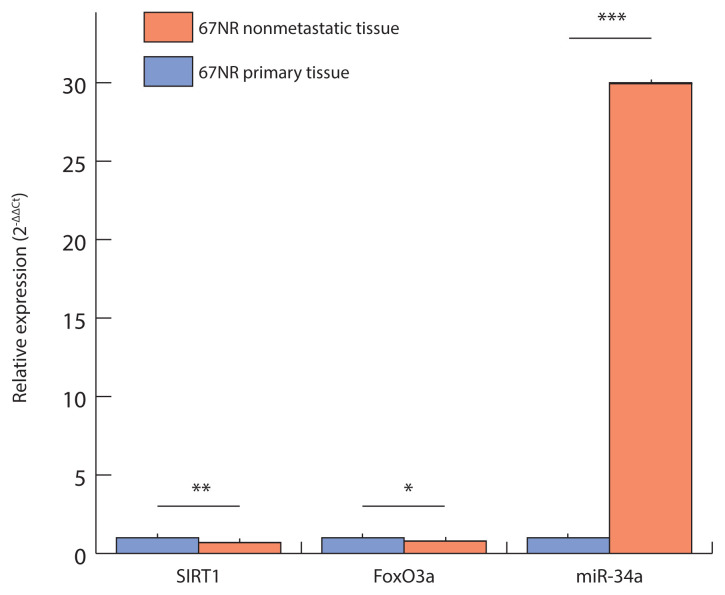
Relative gene expression (2^−ΔΔCt^) of SIRT1, FoxO3a, and miR-34a in 67NR primary and nonmetastatic tumor groups. Expression levels were normalized to 18S rRNA, with the primary group set as 1.0. bars represent mean ± SD values. Asterisks above the bars indicate significant differences between primary and nonmetastatic tissues for each gene (SIRT1: p = 0.005; FoxO3a: p = 0.03; miR-34a: p = 0.0001; independent t-tests).

In contrast, Figure 2 demonstrates that in the 67NR nonmetastatic model, gene expression profiling revealed the opposite pattern: SIRT1 and FoxO3a expression levels were significantly lower in nonmetastatic tumors compared to the corresponding primary tumors (SIRT1 ≈0.7- fold, p = 0.005; FoxO3a ≈0.8-fold, p = 0.03), whereas miR-34a expression was markedly increased—approximately 30-fold higher than in the primary tumor group (p = 0.0001).

These findings underscore the inverse relationship between miR-34a and SIRT1 expression in metastatic tissues, consistent with the hypothesis that miR-34a acts as a negative regulator of SIRT1. Although FoxO3a exhibited relatively modest expression changes across both models, its regulation appears to be context-dependent, potentially influenced by metastatic status and the tumor microenvironment.

Overall, these findings indicate that SIRT1 upregulation and miR-34a downregulation are key molecular features of metastasis, particularly evident in the 4TLM model. The expression profiles shown in Figures 1 and 2 provide valuable insights into the miR-34a/SIRT1/FoxO3a regulatory axis, highlighting the complex and potentially therapeutic significance of these molecules in breast cancer progression.

Our study further examined the regulatory role of miR-34a in modulating FoxO3a signaling during metastatic progression. We confirmed that miR-34a expression was significantly reduced in metastatic tissues, consistent with its well-established tumor-suppressive role [1,7].

Previous studies have shown that overexpression of miR-34a enhances FoxO3a expression and promotes apoptotic responses, consistent with our observation that miR-34a suppresses cancer cell proliferation and metastatic potential through FoxO3a modulation. Notably, these effects are mediated through critical downstream targets, including Notch1, BCL2, and CDK6 [1,9].

Additionally, expression analysis of 67NR tumors revealed that FoxO3a and SIRT1 expression levels were higher in primary tumors than in nonmetastatic tissues, whereas miR-34a expression was markedly reduced in metastatic samples (p < 0.05), further reinforcing its tumor-suppressive role. These observations support a context-dependent model in which SIRT1 functions as a tumor suppressor in nonmetastatic settings but acts as a tumor promoter under metastatic conditions. These findings are in agreement with previous reports, further validating the dual regulatory role of SIRT1 in tumor biology. For instance, Li et al. demonstrated that miR-34a inhibits SIRT1, thereby modulating apoptosis-related pathways involving FoxO3a [1]. Similarly, Zhang et al. reported that decreased miR-34a expression correlates with poor prognosis and reduced overall survival [7], findings that are consistent with our observation of significantly reduced miR-34a levels in metastatic tissues.

The dual role of SIRT1, observed as protective in primary or nonmetastatic tumors but protumorigenic in metastatic settings, may arise from its binding-dependent activity and dynamic responsiveness to the tumor microenvironment. Elevated SIRT1 expression in primary 67NR tumors suggests a tumor-suppressive role, possibly mediated through FoxO3a regulation. Conversely, in metastatic contexts, SIRT1 may facilitate tumorigenesis by promoting cell proliferation, survival, and invasion.

The marked downregulation of FoxO3a in 67NR nonmetastatic tumors, as shown in Figure 2, suggests that the tumor-suppressive functions of FoxO3a are activated under conditions of relatively high miR-34a expression (p < 0.05). The reduced miR-34a expression observed in metastatic tissues may release the inhibitory constraint on SIRT1, thereby promoting a prometastatic phenotype. This increase in SIRT1 activity is believed to suppress apoptosis by modulating the transcriptional activity of its downstream target, FoxO3a, through deacetylation. Such modulation impairs the established role of FoxO3a in inducing cell-cycle arrest and apoptosis in breast cancer cells. Therefore, the role of FoxO3a appears to function as a secondary mechanism dependent on the primary miR-34a–SIRT1 regulatory axis. This refined understanding clarifies the molecular hierarchy within the miR-34a/SIRT1/FoxO3a network and underscores the therapeutic potential of targeting the miR-34a/SIRT1 interaction in metastatic breast cancer. Previous studies have demonstrated that the tumor suppressor p53 transcriptionally regulates miR-34a. Mutations in p53, which are frequently observed in breast cancers, result in diminished miR-34a expression, providing a potential upstream regulatory pathway that explains the mechanism observed in our study. Loss of functional p53 may enhance metastatic potential by impairing miR-34a-mediated activation of FoxO3a and apoptotic signaling pathways. Further exploration of the p53–miR-34a–FoxO3a axis may reveal novel avenues for therapeutic intervention, particularly in tumors harboring TP53 mutations [16]. Future research should aim to delineate the precise molecular interactions among miR-34a, SIRT1, and FoxO3a across different breast cancer subtypes. Although our findings reveal a strong correlation between the expression of miR-34a, SIRT1, and FoxO3a and metastatic status, the present study remains primarily correlative. To establish a definitive causal relationship, future studies should incorporate functional assays, such as miR-34a overexpression, SIRT1 knockdown, or selective SIRT1 inhibition. Understanding these molecular dynamics may reveal context-specific therapeutic targets for antimetastatic interventions. In vivo investigations evaluating the impact of miR-34a modulation on metastatic potential and survival outcomes—particularly within treatment-resistant subtypes such as TNBC—would be especially valuable.

## 4. Limitations

This study has several important limitations that should be acknowledged. First, the sample size of eight mice per group limits both the statistical power and generalizability of the findings, although this number remains acceptable for a preliminary mechanistic investigation. Second, the use of archived cDNA samples may have introduced variability in sample integrity and potential batch effects. Third, this study is correlational in nature; therefore, functional experiments—such as miR-34a overexpression or SIRT1 inhibition—are necessary to establish causality within the observed miR-34a/SIRT1/FoxO3a axis. Finally, the findings are limited to murine models and have not yet been validated in human breast cancer tissues or clinical patient cohorts. Differences between murine models and the human tumor microenvironment may influence the direct translational relevance of these findings.

## 5. Conclusion

In summary, this study delineates a complex regulatory axis involving miR-34a, FoxO3a, and SIRT1, underscoring their context-dependent roles in breast cancer progression. The reduced expression of miR-34a and FoxO3a in metastatic tissues reinforces their well-established tumor-suppressive functions, whereas the dual nature of SIRT1—acting as either a tumor suppressor or promoter depending on cellular context—highlights its significance as a modifiable molecular target. A limitation of this study is the sample size of 16 mice (eight per group), which—although adequate for a focused mechanistic investigation—may limit the statistical power and generalizability of the findings.

These findings suggest that strategic modulation of the miR-34a/SIRT1/FoxO3a pathway may offer promising therapeutic opportunities, particularly for aggressive and treatment-refractory breast cancer subtypes such as triple-negative breast cancer (TNBC). Future therapeutic strategies aimed at restoring miR-34a expression or selectively inhibiting SIRT1 activity in metastatic contexts may prove instrumental in suppressing disease progression and improving patient outcomes.

In light of these findings, future studies should employ functional assays to validate the proposed miR-34a/SIRT1/FoxO3a regulatory axis. Functional experiments employing miR-34a mimics for restoration or siRNA-based/chemical inhibition of SIRT1 in metastatic cell lines would help clarify the causal relationship within this regulatory network. Furthermore, to assess the clinical significance of these findings, validation using human breast cancer tissue microarrays or publicly available datasets such as The Cancer Genome Atlas (TCGA) is crucial—particularly in aggressive subtypes like TNBC. Such studies will lay the groundwork for developing the miR-34a/SIRT1/FoxO3a axis as a potential therapeutic target in metastatic breast cancer.

## Figures and Tables

**Table:** RT-PCR primer sequences used for gene expression analysis.

Genes	Forward	Reverse
FoxO3a	GCTAAGCAGGCCTCATCTCA [13]	TTCCGTCAGTTTGAGGGTCT [13]
miR-34a	CGTCACCTCTTAGGCTTGGA [14]	CATTGGTGTCGTTGTGCTCT [14]
18s (housekeep.)	GGTGCATGGCCGTTCTTA [15]	TCGTTCGTTATCGGAATTAACC [15]

## Footnotes

1 National Cancer Institute (2021). What Is Cancer? [online]. Website https://www.cancer.gov/about-cancer/understanding/what-is-cancer [accessed 10 January 2023].

2 International Agency for Research on Cancer (n.d.). Cancer Today. Global Cancer Observatory. [online]. Website https://gco.iarc.fr/today/en/dataviz/bars-compare-populations?mode=cancer&group_populations=1&sexes=0&populations=900&sort_by=value1&types=0_1&key=total [accessed 15 January 2023].

3 International Agency for Research on Cancer (n.d.-a). Cancer Today. Global Cancer Observatory. [online]. Website https://gco.iarc.fr/today/en/dataviz/pie?mode=cancer&group_populations=1&sexes=2 [accessed 18 March 2023].
